# S‐Nitrosylation of Dexras1 Controls Post‐Stroke Recovery via Regulation of Neuronal Excitability and Dendritic Remodeling

**DOI:** 10.1111/cns.70199

**Published:** 2025-01-03

**Authors:** Zhou Han, Yixuan Song, Cheng Qin, Haihui Zhou, Dan Han, Simin Yan, Huanyu Ni

**Affiliations:** ^1^ Department of Pharmacy, Nanjing Drum Tower Hospital The Affiliated Hospital of Nanjing University Medical School Nanjing Jiangsu China; ^2^ Nanjing Medical Center for Clinical Pharmacy Nanjing Jiangsu China; ^3^ Department of Histoembryology, Genetics and Developmental Biology, Shanghai Key Laboratory of Reproductive Medicine Shanghai Jiao Tong University School of Medicine Shanghai China; ^4^ School of Life Sciences and Chemical Engineering Jiangsu Second Normal University Nanjing China

**Keywords:** dendritic remodeling, functional recovery, ischemic stroke, neuronal excitability, SNO‐Dexras1

## Abstract

**Aims:**

Stroke is a major public health concern leading to high rates of death and disability worldwide, unfortunately with no effective treatment available for stroke recovery during the repair phase.

**Methods:**

Photothrombotic stroke was induced in mice. Adeno‐associated viruses (AAV) were microinjected into the peri‐infarct cortex immediately after photothrombotic stroke. Grid‐walking task and cylinder task were used to assess motor function. Western blotting, Golgi staining, and electrophysiology recordings were performed to uncover the mechanisms.

**Results:**

The ternary complex of neuronal nitric oxide synthase (nNOS), carboxy‐terminal PDZ ligand of nNOS (CAPON) and dexamethasone‐induced ras protein 1 (Dexras1) is structurally beneficial for S‐nitrosylation of Dexras1 (SNO‐Dexras1). In our previous study, uncoupling nNOS‐CAPON interaction by Tat‐CAPON‐12C promoted functional recovery after stroke. Here, we show that ischemia elevated the levels of nNOS‐Dexras1 complex and SNO‐Dexras1 in the peri‐infarct cortex in the days 4–10 after stroke induction, and as excepted, Tat‐CAPON‐12C, a peptide disrupting nNOS‐CAPON interaction, significantly reversed these changes. The above information implies that repressed SNO‐Dexras1 may mediate functional‐promoting effects of Tat‐CAPON‐12C and SNO‐Dexras1 could be the vital molecular substrate for post‐stroke functional recovery in the repair phage. Inhibiting the ischemia‐induced SNO‐Dexras1 by AAV vector‐mediated knockdown of Dexras1 or over‐expression of dominant negative Dexras1 (Dexras1‐C11S) produced sustained recovery of motor function from stroke. In contrast, up‐regulation of SNO‐Dexras1 by over‐expressing Dexras1 worsened stroke outcome. Using electrophysiology recordings, we also observed that silence of Dexras1 in the peri‐infarct cortex increased the spike number and the miniature excitatory postsynaptic currents (mEPSCs) frequency, suggesting enhancement of neuronal excitability. In addition, silence of Dexras1 increased dendritic complexity in cultured neuron and more importantly enhanced dendritic spine density in the peri‐infarct cortex, implying dendritic remodeling.

**Conclusion:**

Thus, inhibition of SNO‐Dexras1 positively regulates post‐stroke functional recovery via enhanced neuronal excitability and dendritic remodeling. Our results identify that SNO‐Dexras1 may serve as a novel target for promoting motor functional restoration from stroke in the delayed phase, shedding light on stroke treatment.

## Graphical abstract

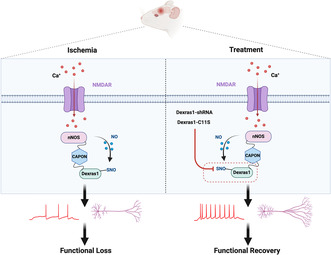

## 1 Introduction

Ischemic stroke remains the leading cause of death and disability in the world [1]. The most common pathological etiology underlying ischemic stroke is the interruption of cerebral blood perfusion induced by atherothrombosis. Although reperfusion therapies, such as thrombolysis and thrombectomy, are theoretically the most effective treatments for acute stroke, their clinical application is largely limited by the narrow therapeutic time window and the risk of hemorrhagic transformation [2]. It has been reported that only less 10% of stroke patients are qualified for these therapies, and even fewer achieve significant therapeutic benefits [2]. Despite extensive studies into neuroprotective treatments for acute stroke, no cytoprotective agent has a definitive therapeutic efficacy in clinical trials. Furthermore, in very recent years, ischemic stroke has increasingly been recognized as a chronic disease [3]. Accordingly, researches have shifted their primary emphasis from neuroprotective strategies aimed at mitigating damage during acute stroke episodes to long‐term neurorestorative approaches focused on facilitating functional recovery in the chronic phase of stroke.

Neuroplasticity, mainly including neuronal excitability, dendritic remodeling, and axonal sprouting, is the core alteration in the peri‐infarct cortex in the repair phase of stroke [4, 5]. Using pharmacological and genetic methods, our team and other researchers have demonstrated that enhanced neuroplasticity accounts for post‐stroke functional recovery [6, 7, 8, 9]. However, there is a lack of drugs available for stroke recovery by promoting neuroplasticity and long‐term therapeutic strategy is still restricted to specific rehabilitation programs, underscoring an urgent need to accelerate the development of pharmacological therapies for stroke recovery.

Dexamethasone‐induced ras protein 1 (Dexras1) is a brain‐enriched member of the Ras family of small monomeric G proteins [10]. As reported, Dexras1 plays critical roles in schizophrenia, anxiety, and NMDA‐ and Aβ‐induced neurotoxicity [11, 12, 13, 14]. Dexras1 binds to neuronal nitric oxide synthase (nNOS) through carboxy‐terminal PDZ ligand of nNOS (CAPON) to form a ternary complex of nNOS‐CAPON‐Dexras1 [15]. This spatial conformation facilitates the S‐nitrosylation modification of the 11th Cysteine residue of Dexras1 by nNOS‐derived nitric oxide (NO), thereby activating Dexras1 [10]. Activated Dexras1 negatively regulates neuronal excitability through inhibiting PKC/Src pathway [16]. Our previous study has revealed nNOS‐CAPON coupling participated in the pathology of stroke in the repair phase, and more importantly, disrupted this interaction strengthened neuroplasticity and thus reversed stroke‐induced function loss [9]. Accordingly, we inferred that Dexras1 may serve the key downstream molecule of nNOS‐CAPON binding and mediate regulation of nNOS‐CAPON interaction in neuroplasticity and behavioral modifications. Therefore, in this study we would explore the roles of Dexras1 in post‐stroke neural repair and its potential mechanisms.

## 2 Materials and Methods

### 2.1 Animals

Male young adult (7–8 weeks) C57BL/6 mice are from Model Animal Research Center of Nanjing University, China. Mice were maintained at controlled temperature (20°C ± 2°C) and housed them (12‐h light–dark cycle) with access to food and water ad libitum. All animal experiments were approved by the Institutional Animal Care and Use Committee (IACUC) of Nanjing University.

### 2.2 Drugs

Tat‐CAPON‐12C/A22D and Tat‐CAPON‐12C were designed and synthesized in our laboratory. All drugs used in electrophysiological experiments were supplied by Sigma or Tocris.

### 2.3 Cell Cultures

Primary cortical neurons were isolated and cultured as described previously with some modifications [17]. Cortex tissues from E16 mouse were dissected and placed in Hank's balanced salt solution without Ca^2+^ and Mg^2+^ (Gibco BRL, Grand Island, NY, USA). Then, the tissues were dissociated in HBSS containing 0.125% trypsin solution for 10 min at 37°C. Subsequently, the tissues were triturated by repeated passage through a constricted Pasteur pipette. The digestion was stopped with DMEM along with 10% heat‐inactivated fetal bovine serum. The dispersed tissues were centrifuged at 200 *g* for 5 min. The pellet was resuspended in a neuron‐defined culture medium, serum‐free neurobasal medium (Gibco), supplemented with B27, 0.5 mM L‐glutamine, 20 penicillin, and 20 IU/mL streptomycin. The cells were then plated onto 1 mm^2^ coverslips coated with poly‐D‐lysine (100 μg/mL) at 3 × 10^4^/cm^2^ for morphological analysis. Half of the medium was replaced with fresh medium every 2–3 d. Cell cultures were maintained in an incubator (Heracell 150; Thermo Fisher Scientific) with a humidified atmosphere of 95% air and 5% CO_2_ at 37°C.

### 2.4 Recombinant Virus Production and Injection

In the present study, all viruses were generated by GeneChem Co. Ltd. (Shanghai, China). The coding sequence of mouse Dexras1 and Dexras1‐C11S were amplified by RT‐PCR. The primers were as follows: for Dexras1, forward: 5′‐GAG GAT CCC CGG GTA CCG GTC GCC ACC ATG AAA CTG GCC GCG ATG‐3′, reverse: 5′‐TCA CCA TGG TGG CGA CCG GAC TGA TGA CAC AGC GCT CCT‐3′. Dexras1 and Dexras1‐C11S had the same primers. Dexras1 and Dexras1‐C11S genes were sub‐cloned into pAAV.CMV.EGFP.3Flag plasmids to produce pAAV.CMV.Dexras1.EGFP.3FLAG, pAAV.CMV.Dexras1‐C11S.EGFP.3FLAG. AAV‐Dexras1‐GFP and AAV‐Dexras1‐C11S‐GFP were produced by transfection of AAV‐293 cells with the above corresponding plasmids, AAV helper plasmid (pHelper), and AAV Rep/Cap expression plasmid (pAAV‐RC). Viral particles were purified by an iodixanol step‐gradient ultracentrifugation method. The genomic titer was 1.2–1.5 × 10^13^ genomic copies per mL determined by quantitative PCR. GFP‐3Flag was fused to the C‐terminus of Dexras1 and Dexras1‐C11S.

The shRNA of Dexras1 was constructed and synthesized by GeneChem Co. Ltd. (Shanghai, China). The target sequence used against mouse Dexras1 was as follows: 5′‐AGG ACT AAT AAT AGG GCA T‐3′. Recombinant adeno‐associated virus AAV‐Dexras1‐shRNA‐GFP was produced by cotransfecting 293 T cells with the pAAV‐RC plasmid and pHelper plasmids using CaCl_2_/HBS solution.

In this study, all AAVs were delivered (1 nL/s, 1 μL) into peri‐infarct cortex immediately after stroke at coordinates (mm): anterior–posterior, 0; medial‐lateral, −1.5; dorsal‐vental, −1.3. The recombinant lentivirus LV‐Dexras1‐shRNA‐GFP was also obtained from GeneChem Co. Ltd. (Shanghai, China) for cell experiments. Cultured neurons were infected with LV‐Dexras1‐shRNA‐GFP or LV‐GFP containing 1.0 × 10^9^ transduction units per ml at d 4 in vitro. The medium was half changed 6 h later and fully changed 24 h later. At d 10 in vitro, neurons were treated for morphological analysis.

### 2.5 Immunofluorescence

The details of immunofluorescence for brain sections and cell cultures have been described previously [9]. Briefly, for immunohistochemistry, under deep anesthesia, mice were transcardially perfused with 0.9% NaCl followed by 4% paraformaldehyde, and their brains were sectioned at 40 μm thickness using a vibratome (VT1000s, Leica). For immunocytochemistry, cortical neurons were fixed using 4% paraformaldehyde for 15 min. Brain slices or cortical neurons were blocked in PBS containing 3% normal goat serum, 0.3% Triton ×‐100, and 0.1% BSA at room temperature for 1 h, followed by incubating with primary antibody rabbit anti‐GFP (Abcam ab290; 1:500) at 4°C overnight. Subsequently, the slices or neurons were incubated with secondary antibodies goat anti‐rabbit Dylight 488 (Jackson ImmunoResearch; 1:400) for 2 h at room temperature. All fluorescence images were captured with a confocal laser‐scanning microscope (LSM700, Zeiss). For morphological analysis, eight neurons from each sample were measured, and the average was regarded as the final value of one sample. Dendritic length and branch number were analyzed. The Sholl analysis for dendritic complexity was carried out by counting the number of dendrites that cross a series of concentric circles, radius from 20 to 200 μm, at 20‐μm intervals from the soma.

### 2.6 Western Blot Analysis

Briefly, the peri‐infarct zone and the equivalent region of cortex from Sham mice were dissected on an ice box and homogenized in RIPA lysis buffer containing proteinase inhibitor. The concentration of total protein was measured by BCA assay. Protein was separated by SDS‐PAGE gel and then transferred to PVDF membrane (Merck Millipore IPVH00010). Membranes were blocked with PBS containing 7.5% non‐fat milk and 0.1% Tween 20 for 1 h at 20°C and subsequently incubated with the following primary antibodies overnight at 4°C: rabbit anti‐nNOS (Thermo Fisher 61–7000; 1:2000), rabbit anti‐Dexras1 (Merck AB15794; 1:2000), rabbit anti‐GFP (Abcam ab290; 1:4000), mouse anti‐Flag (Sigma F1804; 1:4000), and rabbit anti‐Spinophilin (Cell Signaling Technology 13146S; 1:1000). Internal control was performed using mouse anti‐GAPDH (KangChen Bio‐tech KC‐5G4; 1:8000) or rabbit anti‐β‐actin (Sigma A1978; 1:4000). Appropriate horseradish peroxidase‐linked secondary antibodies were used for detection by enhanced chemiluminescence (Pierce).

### 2.7 Co‐Immunoprecipitation

Lysis and co‐immunoprecipitation of tissues were performed as we described previously [14]. Peri‐infarct cortex was lysed in 50 mM Tris–HCl, pH 7.4, buffer containing 150 mM NaCl, 1 mM EDTA‐Na, 1% NP‐40, 0.02% sodium azide, 0.1% SDS, 0.5% sodium deoxycholate, 1% PMSF, 1% aprotinin, 1% leupeptin, and 0.5% pepstatin A. The lysates were centrifuged at 12,000 × g for 15 min at 4°C. The supernatant (200 μL) were incubated with mouse anti‐nNOS (BD Bioscience 610,309; 1:40) or mouse IgG (Merck 12–371; 1:40) for 8–10 h. Protein G‐Sepharose beads (Sigma P4691; 20 μL/sample) and immune complexes were added for incubation overnight at 4°C. Immune complexes were isolated by centrifugation, washed five times with 0.05 M HEPES buffer, pH 7.1, containing 0.15% Triton ×‐100, 0.15 M NaCl, and 0.1 × 10^−3^ M sodium orthovanadate, and bound proteins were eluted by heating at 100°C in loading buffer. Proteins were analyzed by immunoblotting using rabbit anti‐Dexras1 (Merck AB15794; 1:500) or rabbit anti‐nNOS (Thermo Fisher 61–7000; 1:400).

### 2.8 Biotin Switch Assay

This assay was performed as previously described [17]. Briefly, peri‐infarct cortex was homogenized in HEN buffer, pH 8.0, containing 250 mM HEPES, 1 mM EDTA, and 100 mM neocuproine. Free cysteines were blocked for 30 min at 50°C in blocking buffer (HEN buffer plus 2.5% SDS and 0.1% methyl methanethiosulfonate [Sigma 64,306]). Proteins were precipitated with acetone at −20°C and resuspended in 240 μL HENS solution. After adding 30 μL ascorbic acid (20 mM) and 30 μL biotin‐HPDP (Sigma 85,881; 1 mM), proteins were incubated at 30°C for 1 h. Biotinylated proteins resuspended in 250 μL HENS buffer plus 750 μL neutralization buffer and 30 μL prewashed avidin‐affinity resin beads were added for incubation over night at 4°C. The complexes were precipitated and washed five times at 4°C using neutralization buffer containing 600 nM NaCl. Biotinylated proteins were eluted using 30 μL elution buffer and heated at 100°C for 10 min in reducing SDS‐PAGE loading buffer.

### 2.9 Photothrombotic Model of Stroke

Focal cortical ischemia was induced in mice by photothrombosis of cortical microvessels. Briefly, under chloral hydrate anesthesia (400 mg/kg, i.p.), adult male mice were placed in a stereotactic apparatus with the skull exposed through a midline incision, cleared of connective tissue, and dried. A cold light source (World Precision Instruments) attached to an iron washer giving a 2‐mm‐diameter illumination was positioned 1.5 mm lateral from Bregma only in the right hemisphere. Rose Bengal solution (Sigma 330,000; 100 mg/kg, i.p.) was administered. Five minutes later, the brain was under 12,000 lx illumination through the intact skull for 15 min. Through the operation, body temperature was maintained at 37°C ± 0.5°C with a heating pad. Sham‐operated mice only received the equivalent dose of Rose Bengal without illumination.

### 2.10 Behavioral Assessment

*Grid‐walking task* A 12‐mm square wire mesh with a grid area of 32/20/50 cm (length/width/height) was used to conduct the grid‐walking task [8]. A camera was placed beneath the apparatus to allow video footage in order to assess the animals' stepping errors (foot faults). Each mouse was placed individually on top of the elevated wire grid and allowed to freely walk for 5 min. Analysis of video footage was performed offline by raters blind to the treatment groups. The total number of steps for each limb was counted, including foot faults and non‐foot‐fault steps, and a ratio between foot faults and total steps was calculated as follows: number of foot faults/(foot faults + number of non‐foot‐fault steps) × 100. A step was considered a foot fault if it was not providing support and the foot went through the grid hole. Furthermore, if an animal was resting with the grid at the level of the wrist, this was also considered a fault.

*Spontaneous forelimb task* (*cylinder task*) The spontaneous forelimb task encourages the use of forelimbs for vertical wall exploration in a cylinder [8]. When placed in a Plexiglas cylinder (15 cm in height with a diameter of 10 cm), the mouse spontaneously rears to press the cylinder wall with either one or both of its forelimbs. Each mouse was allowed to freely explore for 5 min and videotaped. The video footage was analyzed offline by calculating the time (seconds) during each rear that each animal spent on either the right forelimb, the left forelimb, or on both forelimbs in a slow motion. Only rears in which both forelimbs could be clearly seen were timed. The percentage of time spent on each limb was calculated, and these data were used to derive an asymmetry index, as follows: (percent ipsilateral use) − (percent contralateral use).

### 2.11 Infarct Size Measurement

Nissl staining was used to determine the infarct size at 11 d after stroke as described previously. Briefly, the brains were sectioned at 40 μm thickness using a vibratome (VT1000s; Leica), and sections were immersed in the nissl staining solution at 37°C for 10 min. Then, the images were taken on a Zeiss Axio microscope.

### 2.12 Golgi‐Cox Staining

Fresh brains without perfusion and fixation were used for Golgi‐Cox staining to show subtle morphological alterations in dendritic spines [9]. Golgi‐Cox staining was performed with FD Rapid GolgiStain Kit (FD NeuroTechnologies) according to the manufacturer's protocol. Briefly, the brains were first placed in a mix of solution A and B for 10 d, followed by 2 d in solution C. Then, they were cut on a vibratome (VT1000s; Leica) into 100 μm coronal sections and stained. Images were captured with a Zeiss Axio microscope. For measuring spine density, nine neurons from each sample were measured, and the average was regarded as the final value of one sample.

### 2.13 Electrophysiology

Mice were subjected to photothrombotic stroke and administered of AAV‐Dexras1‐shRNA‐GFP immediately after stroke. At 11 d, under chloral hydrate anesthesia (400 mg/kg, i.p.), the mice were transcardially perfused with ice‐cold cutting solution (in mM, Choline Chloride 110.0, Glucose 20.0, KCl 2.5, CaCl_2_ 0.5, MgCl_2_ 7.0, NaH_2_PO_4_ 1.3, Na_H_CO_3_ 25.0, Na‐ascorbate 1.3, Na‐pyruvate 0.6) and decapitated. Brains were rapidly removed and cut on a vibratome (VT1000s; Leica) into 350 μm cortical slices in ice‐cold cutting solution. For action potentials (AP) recordings, patch‐clamp electrodes were filled with an intracellular solution containing 70 mM potassium gluconate, 70 mM KCl, 2 mM NaCl, 2 mM MgCl_2_, 10 mM HEPES, 1 mM EGTA, 2 mM MgATP, and 0.3 mM Na_2_GTP with the pH adjusted to 7.25–7.30 by KOH (275–285 mOsmol). For miniature excitatory postsynaptic current (mEPSC) recordings, patch‐clamp electrodes were filled with internal pipette solution, containing 132.5 mM cesium gluconate, 17.5 mMCsCl, 2 mM MgCl_2_, 0.5mMEGTA, 10 mM HEPES, 4 mM ATP, 5 mM QX‐314, pH adjusted to 7.25–7.30 by CsOH (275–285 mOsmol); 0.5 mM tetrodotoxin and 20 mM bicuculline were added to block action potentials and GABA_A_ receptor‐mediated currents. The recordings were longer than 5 min. Data were analyzed using Mini Analysis Program 6.0 (Synaptosoft Inc.). Up to 100 events from each neuron were selected at a fixed sampling interval to generate cumulative probabilities.

### 2.14 Statistical Analysis

Data were analyzed using Graph Pad Prism versions 8.0. Shapiro–Wilk normality test was used to evaluate the normality of each dataset. Comparisons among multiple groups were made with one‐way ANOVA followed by Tukey post hoc test. Comparisons between two groups were made with a two‐tailed Student's *t* test. Behavioral data collected at repeating time points were analyzed by two‐way repeated measures ANOVA, followed by Tukey post hoc test. Data were presented as mean ± SEM, and *p* < 0.05 was considered statistically significant. For animal studies, sample size was predetermined by our prior experiments. For other experiments, sample size was obtained with power analysis and sample size software using a significance level of *α* = 0.05 with 90% power to detect statistical differences.

## 3 Results

### 3.1 Stroke Upregulates SNO‐Dexras1 Level in the Repair Phase

**FIGURE 1:**
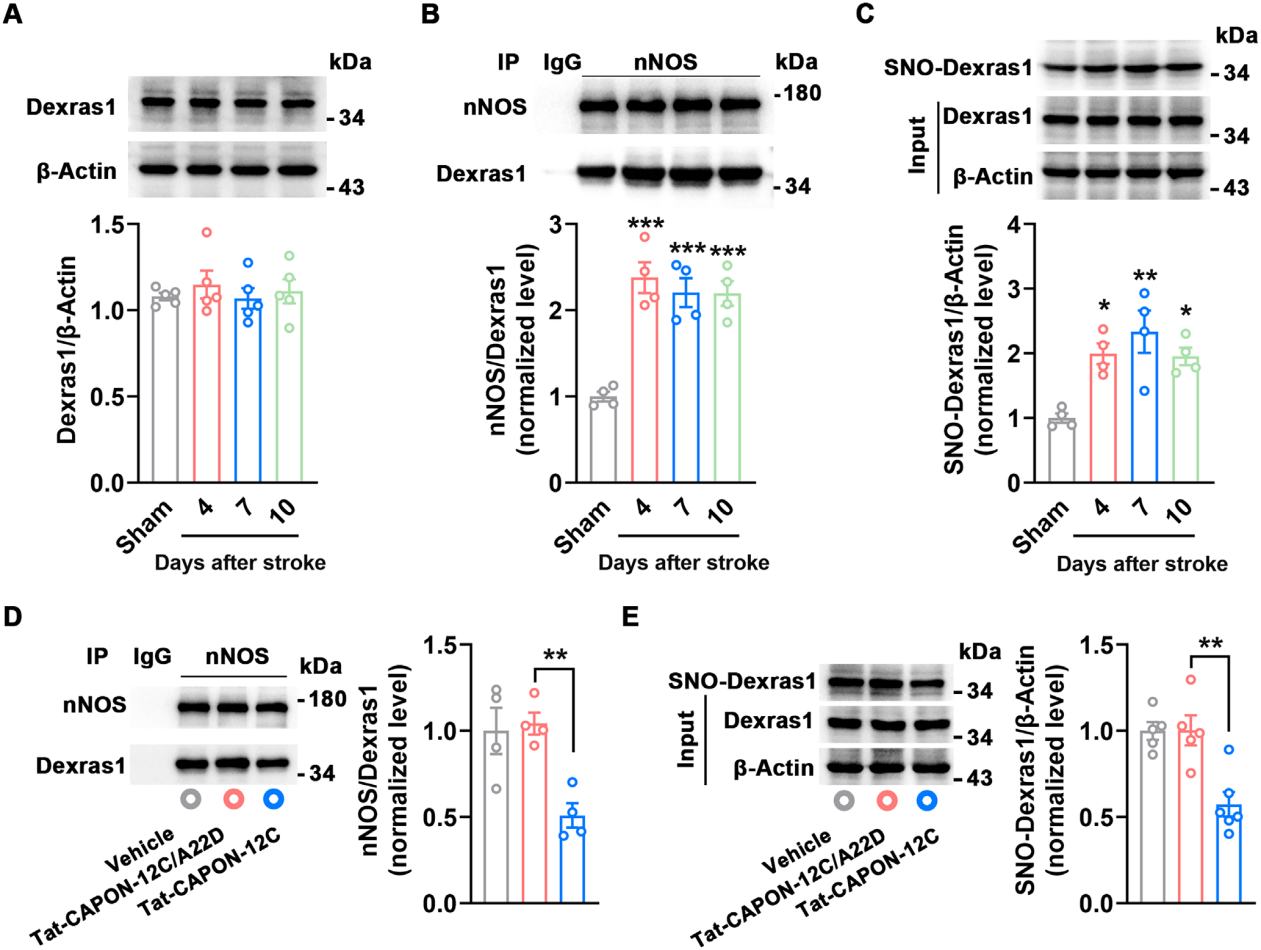
SNO‐Dexras1 participates in the pathological process of stroke. (A) Immunoblots showing time course of Dexras1 expression in the peri‐infarct cortex after stroke (*n* = 5, one‐way ANOVA, Dexras1, *F*_3,16_ = 0.335, *p* = 0.800). (B) Co‐immunoprecipitation showing time course of nNOS‐Dexras1 complex level in the peri‐infarct cortex after stroke (*n* = 4, one‐way ANOVA, *F*_3,12_ = 19.21, ***p < 0.001 vs. Sham). (C) Immunoblots showing time course of SNO‐Dexras1 levels in the peri‐infarct cortex after stroke (*n* = 4, one‐way ANOVA, *F*_3,12_ = 8.405, *p < 0.05, **p < 0.01 vs. Sham). (D) Co‐immunoprecipitation showing nNOS‐Dexras1 complex level at 7 d after stroke in the peri‐infarct cortex of mice treated with Tat‐CAPON‐12C, Tat‐CAPON‐12C/A22D or vehicle 4–7 d after stroke (*n* = 4, one‐way ANOVA, *F*_2,9_ = 9.704, **p = 0.008). (E) Immunoblots showing SNO‐Dexras1 level at 7 d after stroke in the peri‐infarct cortex of mice treated with Tat‐CAPON‐12C, Tat‐CAPON‐12C/A22D or vehicle 4–7 d after stroke (*n* = 5, 5 and 6 for Vehicle, Tat‐CAPON‐12C/A22D, Tat‐CAPON‐12C, respectively, one‐way ANOVA, *F*_2,13_ = 9.704, **p = 0.002).

To test whether Dexras1 participates in the pathology of stroke, we subjected the mice to photothrombotic stroke, measured the Dexras1 level in the peri‐infarct cortex, and unfortunately found that stroke did not affect Dexras1 expression 4–10 days after stroke (Figure 1A). Dexras1 is largely activated by NO‐derived S‐nitrosylation modification [10], and the ternary complex of nNOS, CAPON, and Dexras1 facilitates Dexras1 activation. Our previous study has shown elevation of nNOS‐CAPON coupling during the delayed timeframe of stroke [9]. Intriguingly, we found that stroke enhanced nNOS‐Dexras1 interaction (Figure 1B), suggesting an increase in nNOS‐CAPON‐Dexras1 complex after stroke induction. As excepted, S‐nitrosylation of Dexras1 (SNO‐Dexras1) is observed to be increased in the peri‐infarct cortex during 4–10 days after stroke (Figure 1C). Infusion of Tat‐CAPON‐12C, a peptide disrupting nNOS‐CAPON interaction, into the peri‐infarct cortex via cannulae during 4–10 days after stroke was sufficiently demonstrated to promote neuronal repair and functional recovery from stroke in our previous study [9]. Here, we found that Tat‐CAPON‐12C decreased nNOS‐Dexras1 complex level and thus SNO‐Dexras1 level (Figure 1D,E), implying that SNO‐Dexras1 may mediate stroke recovery‐promoting effects of disruption of nNOS and CAPON. Therefore, SNO‐Dexras1 could participate in the pathological process of stroke, acting as the downstream molecule of nNOS‐CAPON coupling.

### 3.2 Directly Inhibiting Dexras1 S‐Nitrosylation Improves Motor Function

**FIGURE 2:**
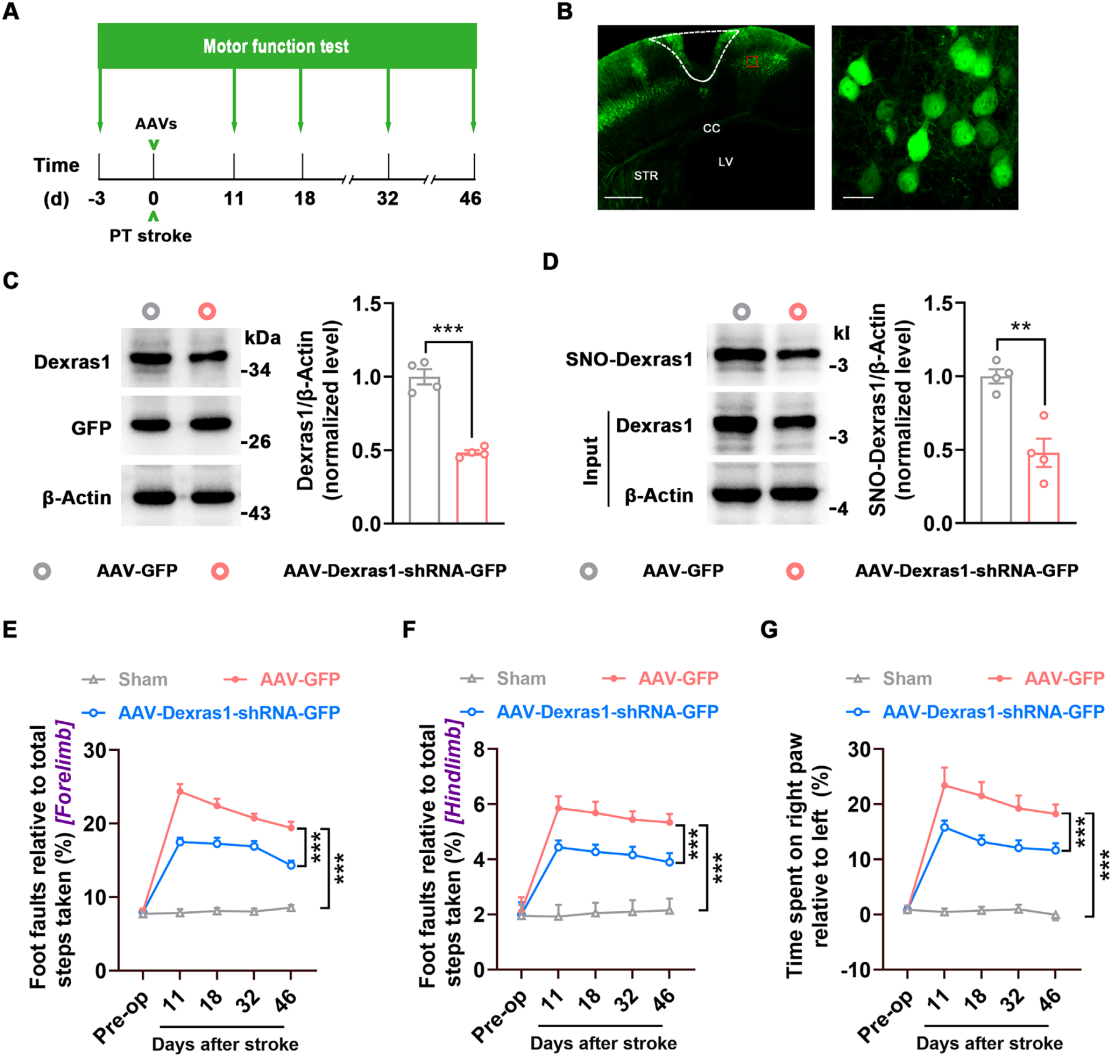
Downregulation of SNO‐Dexras1 level by knockdown of Dexras1 promotes functional recovery after stroke. (A) The diagram showing experimental design for (E–G). AAV‐Dexras1‐shRNA‐GFP (1 μL) or its control was infused into the peri‐infarct cortex of mice immediately after stroke and motor function was assessed at the indicated time. (B) Left, a representative image showing AAV‐infected peri‐infarct cortex at 7 d after stroke. Scale bar, 500 μm. Right, a high‐magnification image from the boxed area in the left image. Scale bar, 20 μm. Dotted white lines indicate the infarct cortex. CC, corpus callosum; LV, lateral ventricles; STR, striatum. (C) Immunoblots (left) and bar graphs (right) showing GFP and Dexras1 levels in AAV‐GFP and AAV‐Dexras1‐shRNA‐GFP‐infected peri‐infarct cortex at 7 d after injection (*n* = 4, two‐tailed Student's *t*‐test, *t*_6_ = 9.472, ***p < 0.001). (D) Immunoblots (left) and bar graphs (right) showing Dexras1 and SNO‐Dexras1 levels in the peri‐infarct cortex at 7 d after AAV‐GFP or AAV‐Dexras1‐shRNA‐GFP injection (*n* = 4, two‐tailed Student's *t*‐test, *t*_6_ = 4.838, **p = 0.003). (E–G) Motor function was assessed at the indicated time. *n* = 10 for Sham, *n* = 14 for others. Two‐way repeated measures ANOVA. (E) Foot faults of the left forelimb in the grid‐walking task (*F*_2,35_ = 182.9, ***p < 0.001). (F) Foot faults of the left hindlimb in the grid‐walking task (*F*_2,35_ = 48.84, ***p < 0.001). (G) Forelimb symmetry in the cylinder task (*F*_2,35_ = 52.47, ***p < 0.001).

**FIGURE 3:**
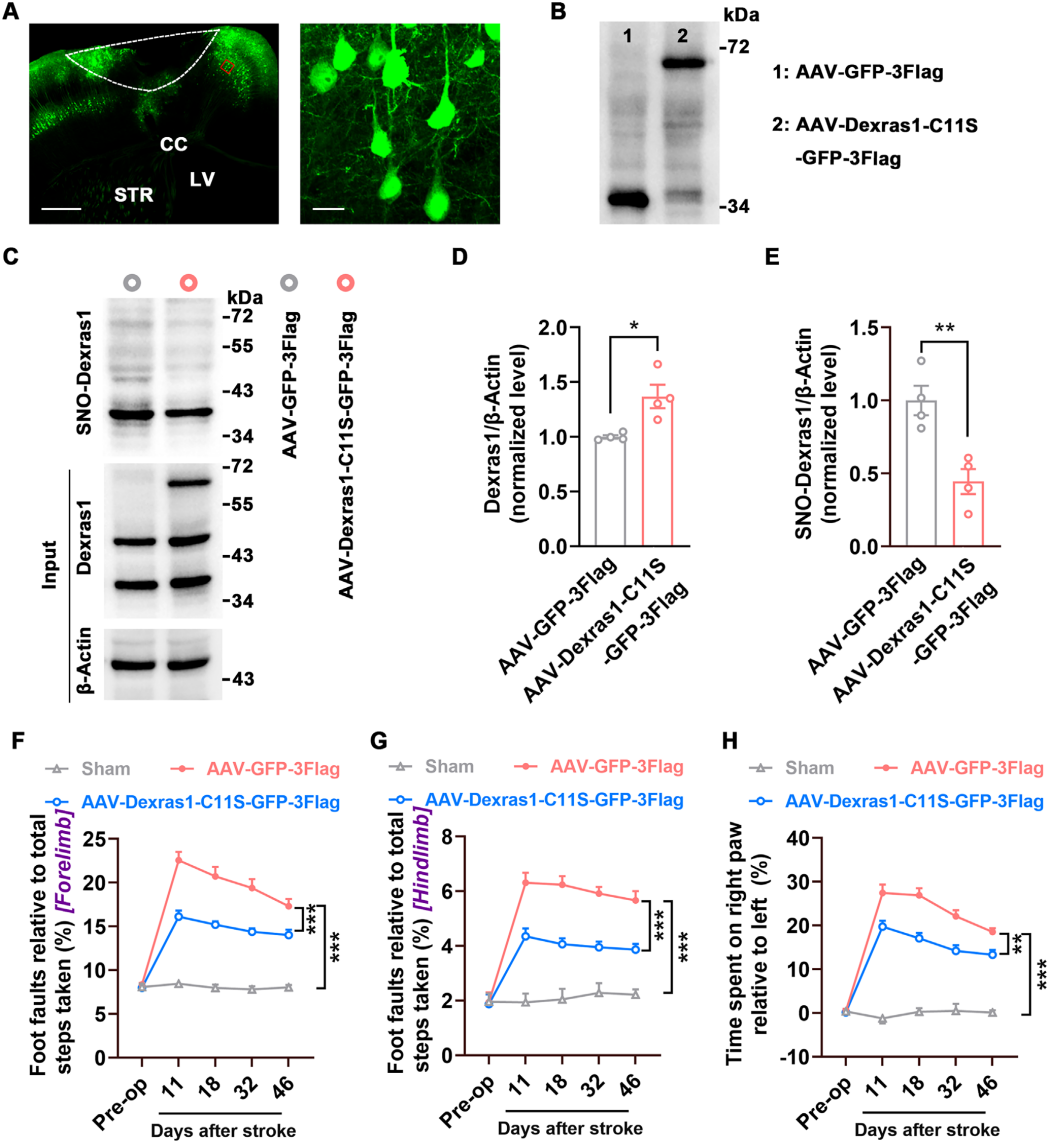
Overexpressing Dexras1‐C11S, a dominant negative Dexras1, decreases SNO‐Dexras1 level and promotes functional recovery after stroke. AAV‐Dexras1‐C11S‐GFP‐3Flag (1 μL) or its control was infused into the peri‐infarct cortex of mice immediately after stroke and motor function was assessed at 11, 18, 32, 46 d after stroke. (A) Left, a representative image showing AAV‐infected peri‐infarct cortex at 7 d after stroke. Scale bar, 500 μm. Right, a high‐magnification image from the boxed area in the left image. Scale bar, 20 μm. Dotted white lines indicate the infarct cortex. CC, corpus callosum; LV, lateral ventricles; STR, striatum. (B) Immunoblots showing GFP‐3Flag and Dexras1‐C11S‐GFP‐3Flag levels in AAV‐GFP‐3Flag and AAV‐Dexras1‐C11S‐GFP‐3Flag‐infected peri‐infarct cortex at 7 d after injection. (C–E) Immunoblots (C) and bar graphs showing Dexras1 (D) and SNO‐Dexras1 (E) levels in the peri‐infarct cortex at 7 d after AAV‐GFP‐3Flag or AAV‐Dexras1‐C11S‐GFP‐3Flag injection. (*n* = 4, two‐tailed Student's *t*‐test, for D, *t*_6_ = 3.427, *p = 0.014; for E, *t*_6_ = 4.199, **p = 0.006). (F–H) Motor function was assessed at the indicated time. *n* = 10 for Sham, *n* = 13 for others. Two‐way repeated measures ANOVA. (F) Foot faults of the left forelimb in the grid‐walking task (*F*_2,33_ = 101.8, ***p < 0.001). (G) Foot faults of the left hindlimb in the grid‐walking task (*F*_2,33_ = 88.42, ***p < 0.001). (H) Forelimb symmetry in the cylinder task (*F*_2,33_ = 194.5, ***p < 0.001).

To determine whether SNO‐Dexras1 affects functional recovery from stroke, we generated an AAV vector containing shRNA of Dexras1 (AAV‐Dexras1‐shRNA‐GFP) and its control AAV‐GFP, infused them into the peri‐infarct cortex of mice immediately after stroke, and detected motor function at 11, 18, 32, and 46 d after stroke (Figure 2A). AAV‐Dexras1‐shRNA‐GFP effectively infected the peri‐infarct cortex (Figure 2B), robustly decreased the levels of Dexras1 and SNO‐Dexras1(Figure 2C,D), and significantly ameliorated stroke‐induced impairment of motor function 11–46 d after stroke (Figure 2E–G). To further test whether SNO‐Dexras1 negatively regulates stroke recovery, we constructed a dominant negative Dexras1 in which cysteine‐11 of Dexras1 is replaced with serine (Dexras1‐C11S), since cysteine‐11 is responsible for the nNOS‐mediated activation of Dexras1 [10]. We inserted the Dexras1‐C11S to an AAV vector tagged with enhanced GFP and 3Flag (AAV‐Dexras1‐C11S‐GFP‐3Flag). AAV‐Dexras1‐C11S‐GFP‐3Flag effectively infected the peri‐infarct cortex (Figure 3A), produced Dexras1‐C11S‐GFP‐3Flag fusion protein (Figure 3B), significantly increased Dexras1 level (including wild‐type Dexras1 and Dexras1‐C11S) (Figure 3C,D), and reduced SNO‐Dexras1 (Figure 3C,E) at 7 d after the infection. Importantly, AAV‐Dexras1‐C11S‐GFP‐3Flag significantly improved motor function 11–46 d after stroke, compared to AAV‐GFP‐3Flag (Figure 3F–H). Thus, inhibition of Dexras1 S‐nitrosylation promotes functional recovery from stroke.

### 3.3 Amplification of Dexras1 S‐Nitrosylation Hinders Functional Recovery

**FIGURE 4:**
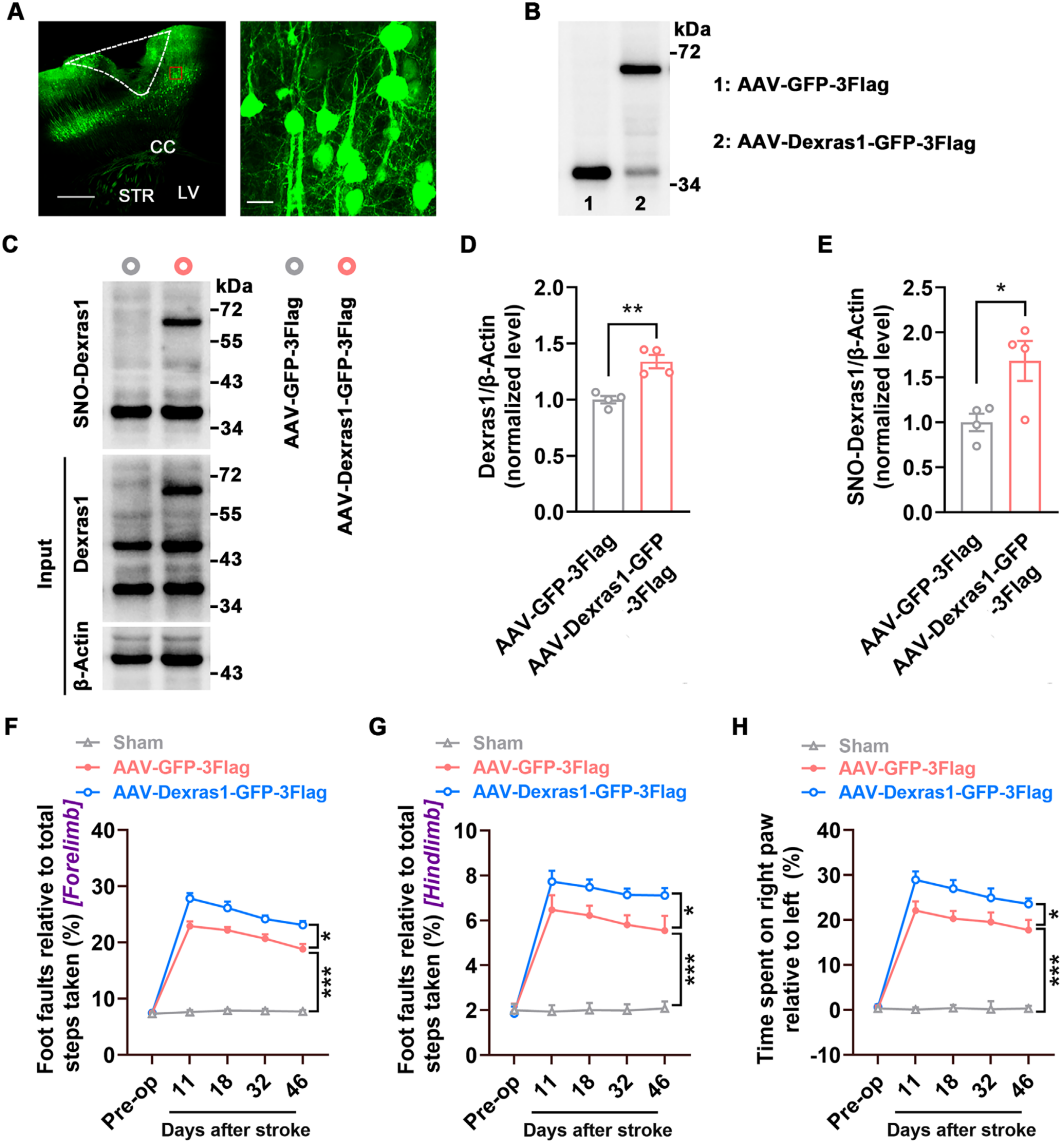
Amplification of Dexras1 S‐nitrosylation by Dexras1 overexpression worsened stroke outcome. AAV‐Dexras1‐GFP‐3Flag (1 μL) or its control was microinjected into the peri‐infarct cortex of mice immediately after stroke and motor function was assessed at 11, 18, 32, 46 d after stroke. (A) Left, a representative image showing AAV‐infected peri‐infarct cortex at 7 d after stroke. Scale bar, 500 μm. Right, a high‐magnification image from the boxed area in the left image. Scale bar, 20 μm. Dotted white lines indicate the infarct cortex. CC, corpus callosum; LV, lateral ventricles; STR, striatum. (B) Immunoblots showing GFP‐3Flag and Dexras1‐GFP‐3Flag levels in AAV‐GFP‐3Flag and AAV‐Dexras1‐GFP‐3Flag‐infected peri‐infarct cortex at 7 d after injection. (C–E) Immunoblots (C) and bar graphs showing Dexras1 (D) and SNO‐Dexras1 (E) levels in the peri‐infarct cortex at 7 d after AAV‐GFP‐3Flag or AAV‐Dexras1‐GFP‐3Flag injection (*n* = 4, two‐tailed Student's *t*‐test, for D, *t*_6_ = 5.007, **p = 0.002; for E, *t*_6_ = 2.816, *p = 0.031). (F–H) Motor function was assessed at the indicated time. *n* = 10 for Sham, *n* = 13 for others. Two‐way repeated measures ANOVA. (F) Foot faults of the left forelimb in the grid‐walking task (*F*_2,33_ = 235.3, *p = 0.02, ***p < 0.001). (G) Foot faults of the left hindlimb in the grid‐walking task (*F*_2,33_ = 92.22, *p = 0.04, ***p < 0.001). (H) Forelimb symmetry in the cylinder task (*F*_2,33_ = 93.31, *p = 0.03, ***p < 0.001).

To investigate whether enhancing Dexras1 S‐nitrosylation hinders functional recovery from stroke, we generated an AAV vector expressing Dexras1 (AAV‐Dexras1‐GFP‐3Flag) and its control AAV‐GFP‐3Flag, infused them into the peri‐infarct cortex of mice immediately after stroke and found that AAV‐Dexras1‐GFP‐3Flag effectively infected the peri‐infarct cortex (Figure 4A), produced Dexras1‐GFP‐3Flag fusion proteins (Figure 4B), and significantly enhanced levels of Dexras1 (including endogenous and exogenous Dexras1) and SNO‐Dexras1 at 7 d after the infection (Figure 4C–E). More importantly, AAV‐Dexras1‐GFP‐3Flag worsened the stroke‐induced functional impairment 11–46 d after stroke, compared to AAV‐GFP‐3Flag (Figure 4F–H), further implicating Dexras1 S‐nitrosylation in functional impairment in the delayed phase after stroke.

### 3.4 Repressing Dexras1 S‐Nitrosylation Enhances Neuronal Excitability

**FIGURE 5:**
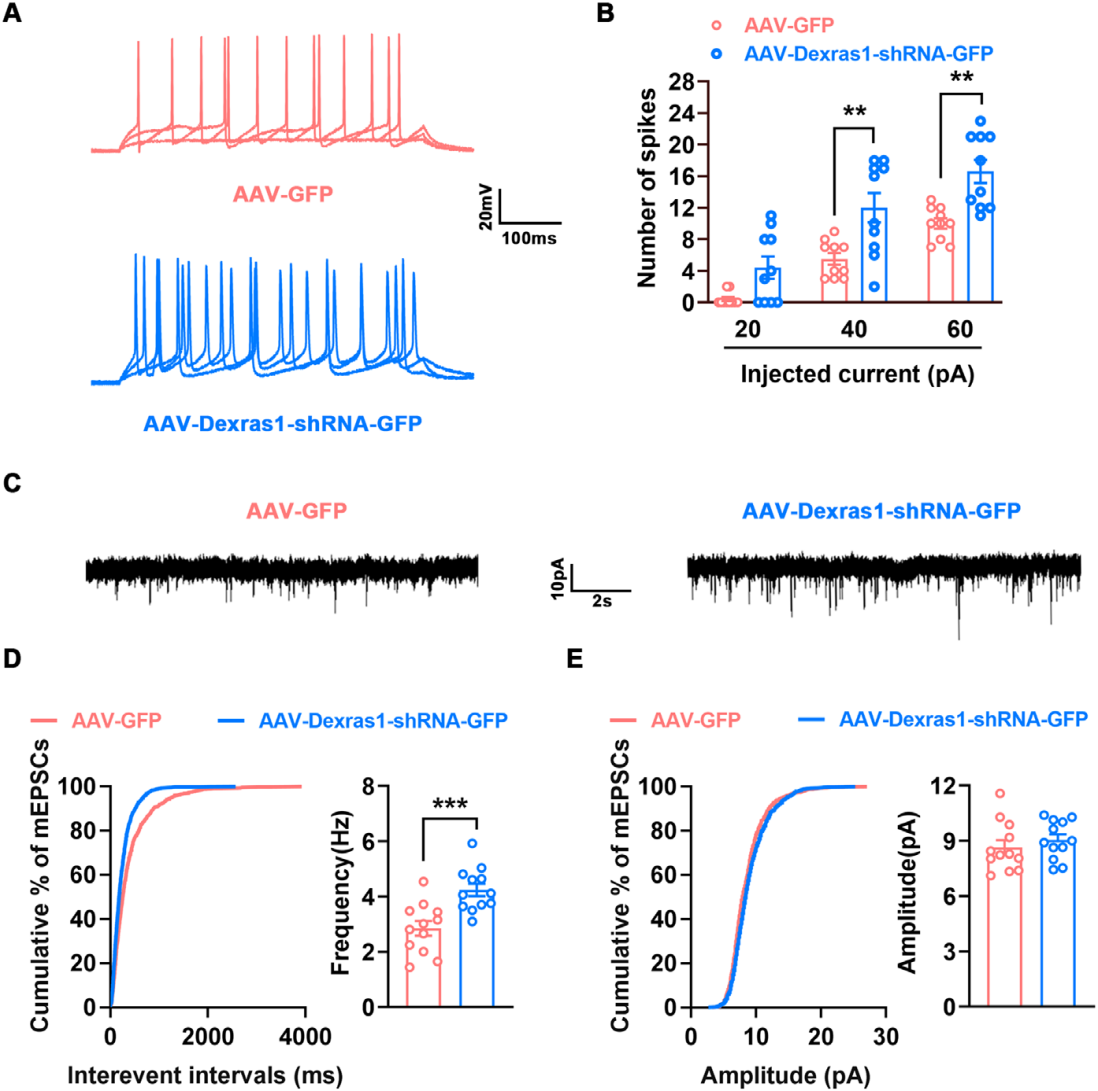
Downregulation of SNO‐Dexras1 level by knockdown of Dexras1 enhances neuronal excitability in the peri‐infarct cortex. AAV‐Dexras1‐shRNA‐GFP (1 μL) or its control was micro‐infused into the peri‐infarct cortex immediately after stroke, and electrophysiology recordings were performed at 11 d after stroke. (A, B) Representative AP traces (A) and AP number (B) evoked by step current injections of 20, 40, 60 pA in peri‐infarct pyramidal neurons infected by AAV‐GFP and AAV‐Dexras1‐shRNA‐GFP (*n* = 10, two‐way ANOVA, *F*_1,54_ = 33.67, **p < 0.01). (C–E) Representative mEPSCs traces (C), mEPSCs frequency (D) and mEPSCs amplitude (E) in peri‐infarct pyramidal neurons from mice of indicated groups (*n* = 12, two‐tailed Student's *t*‐test, *t*_22_ = 4.016, ***p < 0.001).

Accumulating evidence supports that neuronal excitability in the peri‐infarct cortex is crucial for stroke recovery [9, 18]. To test whether Dexras1 S‐nitrosylation regulates neuronal excitability, we infused AAV‐Dexras1‐shRNA‐GFP into the peri‐infarct cortex of mice immediately after stroke and performed electrophysiology experiments at 11 d after stroke. Whole‐cell current clamp was used to detect the firing properties of peri‐infarct pyramidal neurons, and more action potentials were recorded in AAV‐Dexras1‐shRNA‐GFP‐infected neurons than those in AAV‐GFP‐infected neurons in the peri‐infarct cortex (Figure 5A,B), indicating neuronal excitability enhancement after repressed SNO‐Dexras1 by Dexras1 knockdown. Moreover, whole‐cell voltage clamp recordings were performed to assess miniature excitatory postsynaptic currents (mEPSCs) in the peri‐infarct pyramidal neurons. The results showed that AAV‐Dexras1‐shRNA‐GFP remarkedly increased mEPSCs frequency and had no impact on mEPSCs amplitude (Figure 5C–E), suggesting the potential involvement of SNO‐Dexras1 in the regulation of presynaptic neurotransmitter release. Taken together, our data strongly demonstrated that inhibition of SNO‐Dexras1 increases neuronal excitability in the peri‐infarct cortex.

### 3.5 Repressing Dexras1 S‐Nitrosylation Promotes Dendritic Remodeling

**FIGURE 6:**
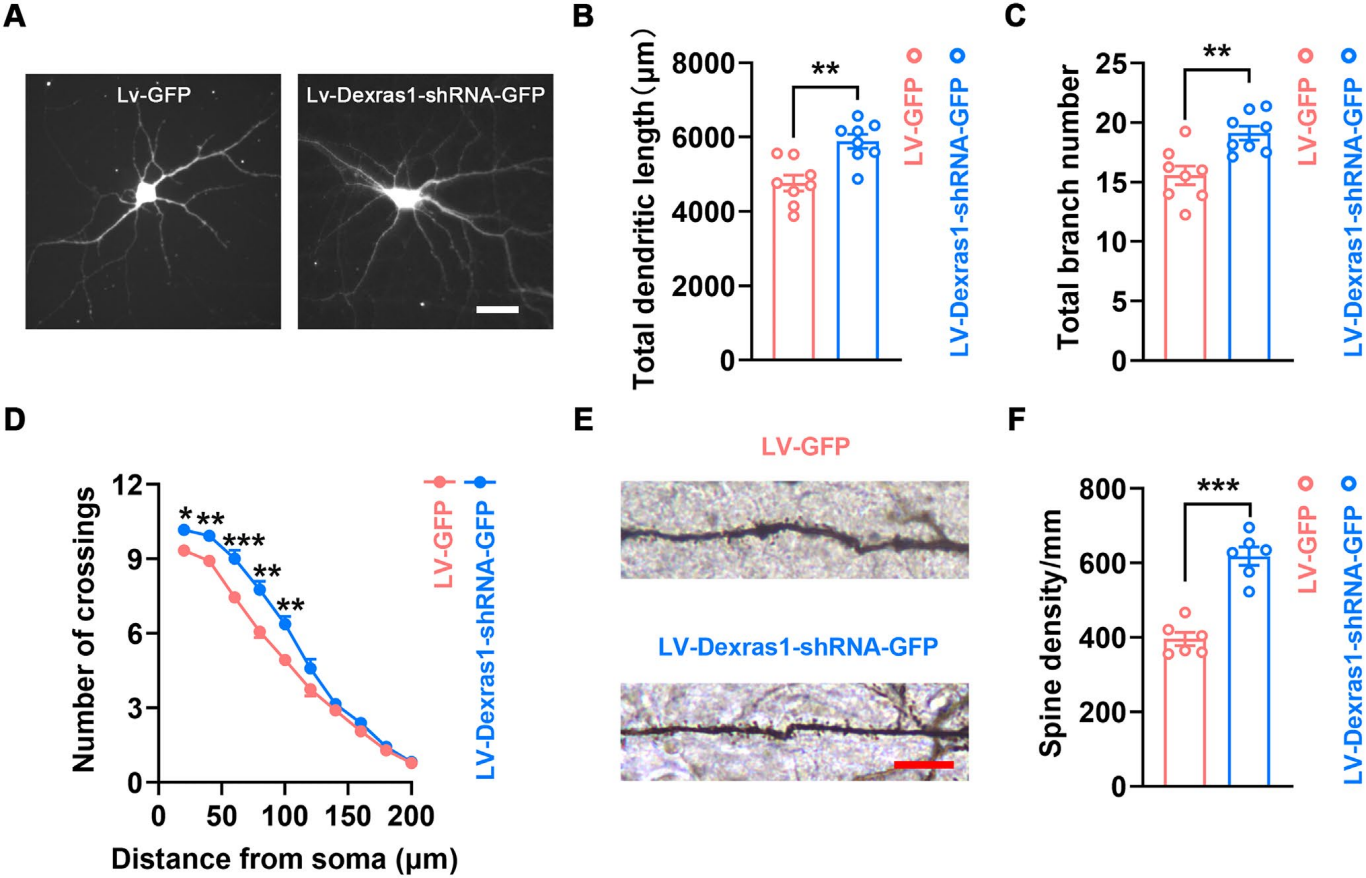
Downregulation of SNO‐Dexras1 level by knockdown of Dexras1 promotes dendritic remodeling. (A) Representative images showing dendrites of the cultured cortical neurons treated with LV‐GFP, LV‐Dexras1‐GFP or LV‐Dexras1‐C11S‐GFP. Scale bar, 40 μm. (B, C) Quantification of total dendritic length (B), branch number (C) and dendritic complexity (D) of neurons infected by LV‐GFP and LV‐Dexras1‐shRNA‐GFP, respectively (*n* = 8, 8 neurons per sample; two‐tailed Student's *t*‐test, for B, *t*_14_ = 3.938, **p = 0.002, for C, *t*_14_ = 3.653, **p = 0.003, for D, *p < 0.05, **p < 0.01, ***p < 0.001). (E, F) Representative images with Golgi‐Cox staining (E) and bar graph (F) showing the dendritic spine density of neurons in the peri‐infarct cortex at 11 d after stroke (*n* = 6, 9 neurons per sample, two‐tailed Student's *t*‐test, *t*_10_ = 7.295, ***p < 0.001). Scale bar, 10 μm.

Given the reports that dendritic remodeling is the common form of structural neuroplasticity and promotes stroke recovery [19, 20], we explored whether SNO‐Dexras1 regulates dendritic remodeling. Primary cortical neurons infected by LV‐Dexras1‐shRNA‐GFP showed significantly increased total process length, total branching number, and dendritic complexity (Figure 6A–D), implicating SNO‐Dexras1 in regulating structural neuroplasticity. To further assess dendritic remodeling in vivo, we infused AAV‐Dexras1‐shRNA‐GFP or its control AAV‐GFP into the peri‐infarct cortex immediately after stroke induction and performed Golgi‐Cox staining at 11 d after stroke. Pyramidal neurons infected by AAV‐Dexras1‐shRNA‐GFP in the peri‐infarct cortex displayed increased dendritic spine density (Figure 6E,F). Besides, we also found that AAV‐Dexras1‐shRNA‐GFP increased the expression of Spinophilin, an important regulator for neuroplasticity, in the peri‐infarct cortex (Figure S1), probably providing a favorable microenvironment for dendritic remodeling. Collectively, these results strongly verified that inhibition of SNO‐Dexras1 promotes dendritic remodeling in the peri‐infarct cortex.

### 3.6 Repressing Dexras1 S‐Nitrosylation Has No Impact on Neuron Survival or Infarct Volume

**FIGURE 7:**
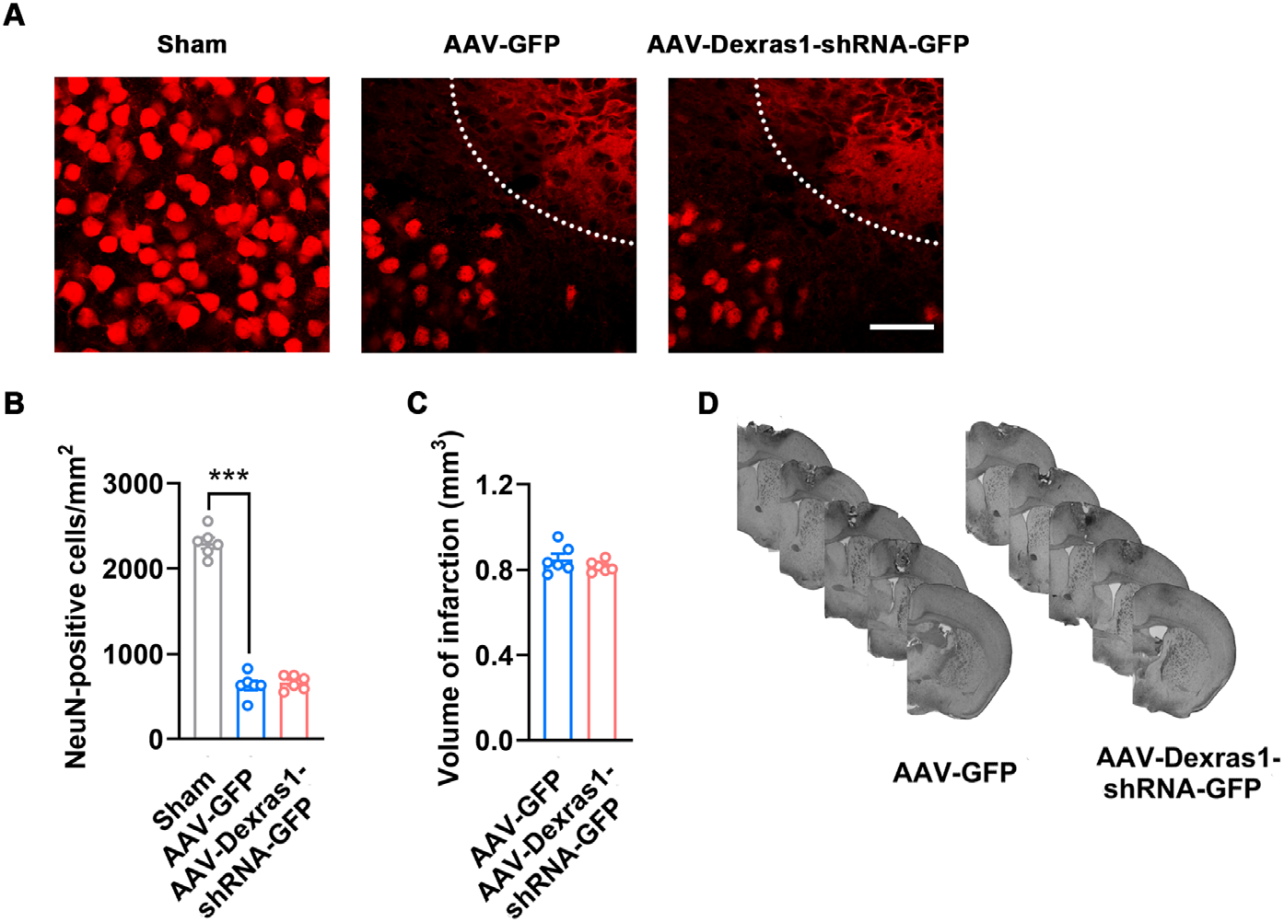
Downregulation of SNO‐Dexras1 level by knockdown of Dexras1 does not affect neuronal survival or infarct size. AAV‐Dexras1‐shRNA‐GFP (1 μL) or its control was micro‐infused into the peri‐infarct cortex immediately after stroke. NeuN and Nissl staining were performed at 11 d after stroke. (A, B) Representative images (A) and bar graph (B) showing the number of surviving neurons in the peri‐infarct cortex at 11 d after stroke. Scale bar, 30 μm. Dotted white lines indicate the infarct cortex. (*n* = 6, one‐way ANOVA, *F*_2,15_ = 319.5, ***p < 0.001). (C, D) Bar graph (C) and representative Nissl‐stained coronal brain sections (D) showing infarct volume (*n* = 6).

In this study, the manipulation of SNO‐Dexras1 level is achieved by the utilization of AAV vectors for the overexpression of Dexras1 or its mutant Dexras1‐C11S, as well as the targeted knockdown of Dexras1, which needs several days to yield substantial effects. Thus, in theory, neuroprotection could not account for stroke recovery in the context of this study. To address this issue, we measured the number of surviving neurons by immunohistochemistry with the antibody against NeuN and infarct size by Nissl staining and found Dexras1 knockdown did not affect surviving neurons in the peri‐infarct cortex or infarct size (Figure 7A–D).

## 4 Discussion

The main findings of this study are that manipulation of Dexras1 S‐nitrosylation in the delayed phase could be a viable strategy for promoting functional recovery from stroke. Given the facts that neurorehabilitation measures are the sole means of promoting stroke recovery in the repair period of stroke [8], with no available medication treatment, our findings in this study provide new insights into stroke recovery.

The consensus has been reached that stroke induced reorganization not only in the peri‐infarcts of ipsilesional hemisphere but also in the contralesional hemisphere [21, 22, 23], suggesting the whole brain rewiring during stroke recovery period. Rodent models of stroke have demonstrated that the progressive changes of neuroplasticity that take place in peri‐infarct neurons [3], and that some of these changes, for examples, axonal sprouting, neuronal excitability and dendritic remodeling, largely correlate with functional recovery from stroke [4, 5, 24]. However, the roles of Dexras1 S‐nitrosylation in regulation of axonal sprouting have not been explored in this study. In addition, neurogenesis also represents an important form of neural repair following stroke [3, 25, 26]. There is a report that Dexras1 regulates neurogenesis [27]. Accordingly, we speculate that Dexras1 S‐nitrosylation may play a role in stroke recovery by regulating neurogenesis. However, in this study, we used photothrombotic stroke to induce local cortical ischemia. It has been reported that photothrombotic stroke results in fewer neuroblasts migrating to the peri‐infarct cortex, with only a negligible number surviving [3, 28], due to the substantial distance between the stroke site and the subventricular zone. Thus, neurogenesis is unlikely to be the primary mechanism responsible for functional restoration after photothrombotic stroke.

The data from humans and rodent models of stroke imply that enhancing neuronal excitability in the peri‐infarct cortex in the delayed phase could be a strategy for stroke recovery [8, 18, 29]. Neuronal excitability enhancement approaches, including anodal direct current stimulation, transcranial magnetic stimulation, optogenetic stimulation, and pharmacogenetic treatments, promoted motor and sensory circuits remapping and thus improved motor performance after stroke [29, 30, 31]. In line with these reports, we also found that suppression of SNO‐Dexras1 by Dexras1 knockdown increased spike numbers of action potential and the frequency of mEPSCs in the infarct surviving neurons, indicating enhanced neuronal excitability. It is noteworthy that elevating excitability in the acute phase may exacerbate excitotoxicity and thus worsen the stroke outcomes. As reported, tonic current inhibitor L655708 and excitatory signaling potentiator CX1837 increased infarct volume when administered in the acute phase of stroke [18, 32]. It has been reported that Dexras1 negatively regulates neuroprotection against NMDA‐induced excitotoxicity and experimental optic neuritis via modulation of iron chelation [11, 33]. However, neuroprotection is a key mechanism in the acute period of stroke. Numerous reports have indicated that no neuroprotective effects were observed when treatments were administered during the delayed phase of stroke [9, 18, 32]. Consistent with these reports, our study also demonstrated that suppressing the level of SNO‐Dexras1 by AAV‐mediated knockdown of Dexras1 in the repair phase did not confer neuroprotection, as evidenced by comparable number of surviving neurons in the peri‐infarct cortex and similar infarct volumes.

Cerebral ischemia rapidly leads to dendritic branching breaks and dendritic spine loss, followed by a slow and limited period of dendritic remodeling [19]. Dendritic remodeling is regarded as an important form of spontaneous repair from stroke, and promoting this process in the delayed phase may be beneficial for stroke recovery [9, 20]. In this study, we found that repressing SNO‐Dexras1 by Dexras1 knockdown increased total process length, total branching number, and dendritic complexity in primary cultured neurons. More importantly, in PT stroke, AAV‐Dexras1‐shRNA‐GFP upregulated the dendritic spine density, compared to AAV‐GFP.

**FIGURE 8:**
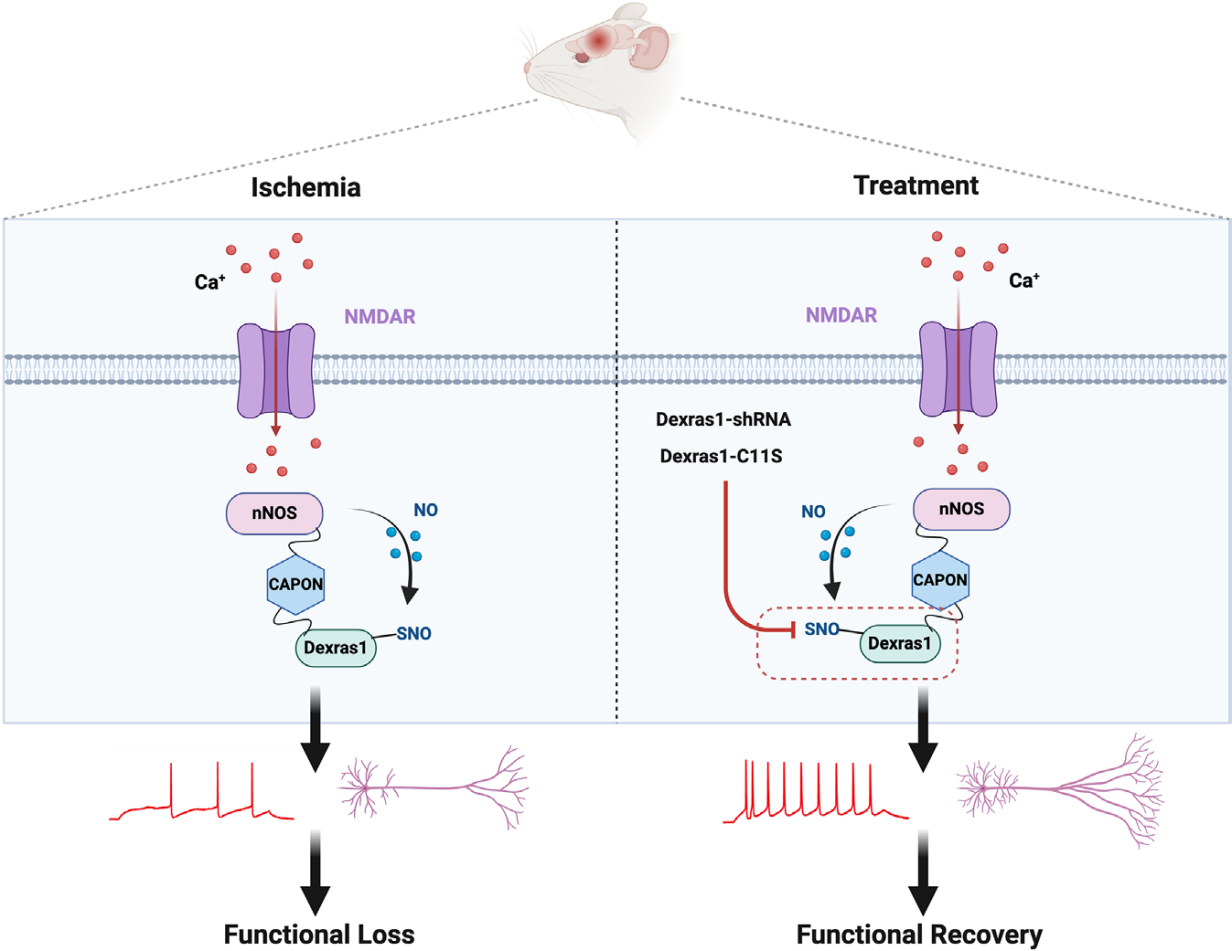
A model showing suppression of SNO‐Dexras1 promotes functional recovery from stroke via strengthened neuronal excitability and dendritic remodeling.

In sum, SNO‐Dexras1 is upregulated in the peri‐infarct cortex in the delayed phase of stroke. Suppression of SNO‐Dexras1 promotes functional recovery from stroke via strengthened neuronal excitability and dendritic remodeling (Figure 8). Our results reveal that SNO‐Dexras1 could serve as the target for stroke recovery.

## Author Contributions

H.N., S.Y., D.H., and Z.H. initiated, designed the study, and wrote the manuscript. Z.H. and Y.S. conducted behavioral experiments, electrophysiological experiments, and data analysis. H.Z. and C.Q. performed molecular biology experiments. All authors approved the manuscript prior to submission.

## Conflicts of Interest

The authors declare no conflicts of interest.

## Supporting information


Figure S1.

Data S1.

## Data Availability

The data support the findings of the current study are available from the corresponding author upon reasonable request.
