# Supplementary material for: S‐Nitrosylation of Dexras1 Controls Post‐Stroke Recovery via Regulation of Neuronal Excitability and Dendritic Remodeling
Source: CNS Neurosci Ther. 2025 Jan 3;31(1):e70199. doi: 10.1111/cns.70199 (PMC11696243; doi:10.1111/cns.70199)
Supplement: Supplementary file 1 — Figure S1. [file CNS-31-e70199-s002.docx]

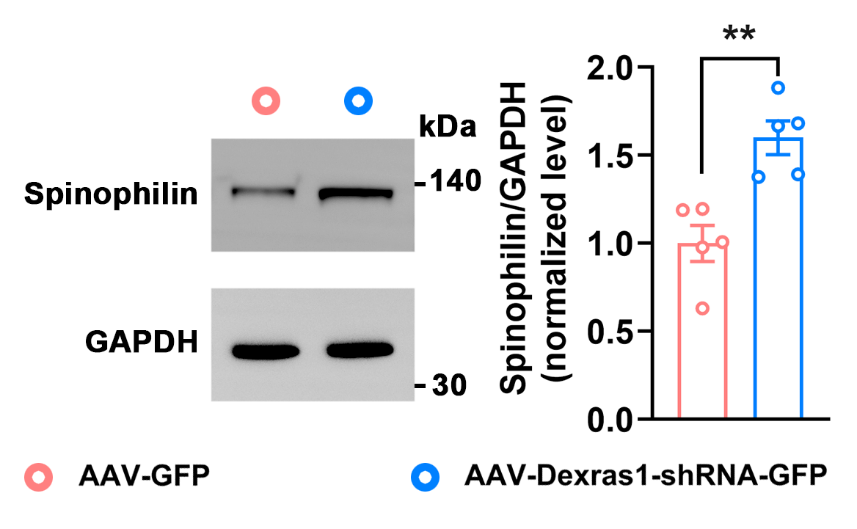


**Figure S1 Downregulation of SNO-Dexras1 level by knockdown of Dexras1 enhances the expression of Spinophilin.** AAV-Dexras1-shRNA-GFP or its control AAV-GFP was infused into the peri-infarct cortex immediately after stroke induction. Immunoblots (left) and bar graph (right) showing the expression of Spinophilin in the peri-infarct cortex at 11 d after stroke (n = 5, *t*_14_ = 4.266, ***P* = 0.003).
