# Supplementary material for: S‐Nitrosylation of Dexras1 Controls Post‐Stroke Recovery via Regulation of Neuronal Excitability and Dendritic Remodeling
Source: CNS Neurosci Ther. 2025 Jan 3;31(1):e70199. doi: 10.1111/cns.70199 (PMC11696243; doi:10.1111/cns.70199)

Full unedited blots for Figure 1A-E, and 2C-D.

Figure 1A

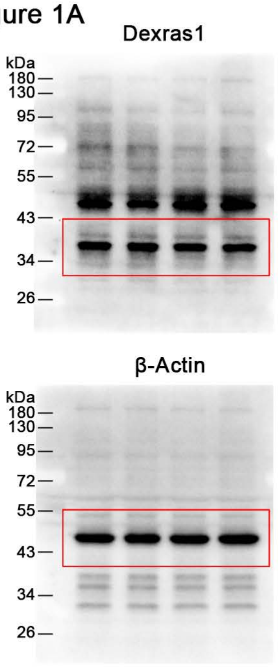

Figure1B

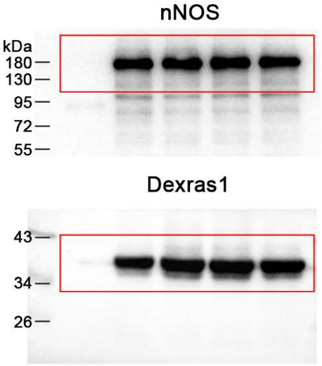

Figure 1C

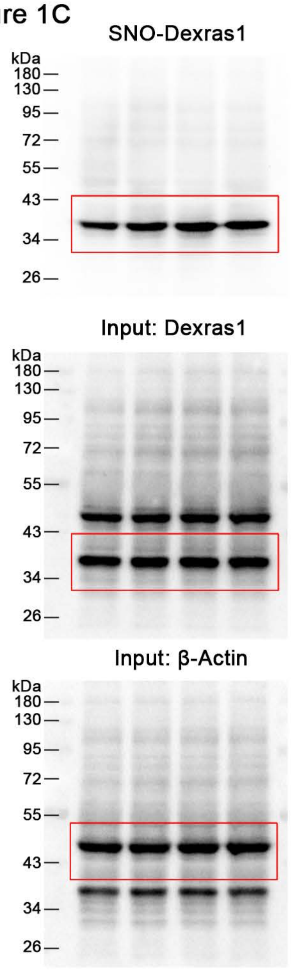

Figure 1D

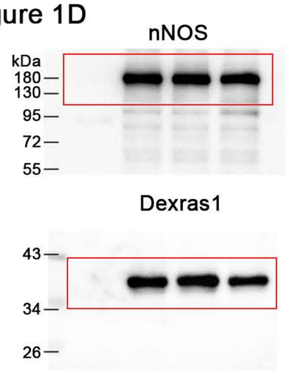

Figure 1E

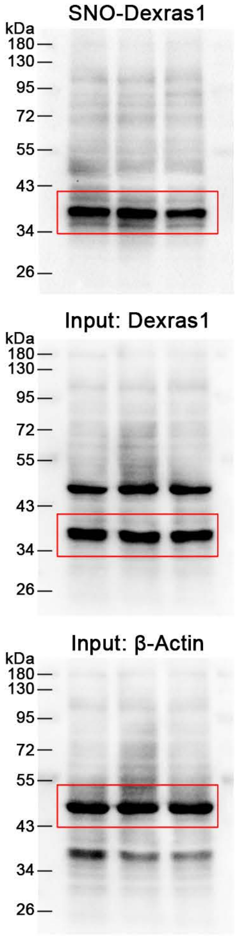

Figure 2C

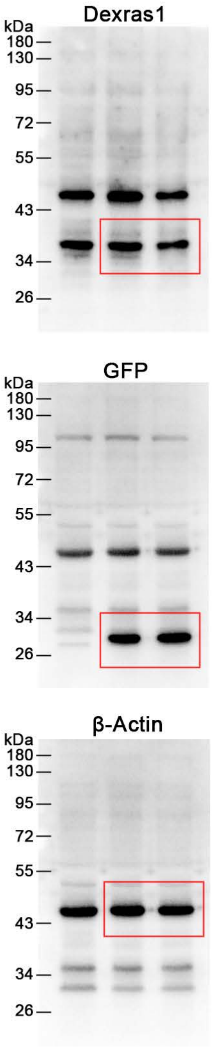

Figure 2D

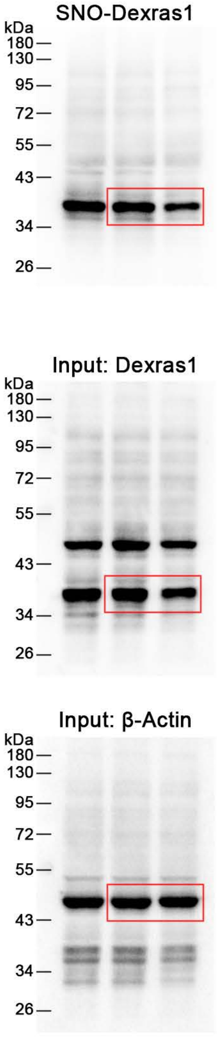

Full unedited blots for Figure 3B-C, 4B-C and S1.

Figure 3B

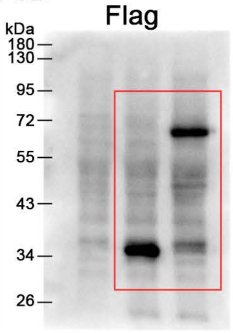

Figure 3C

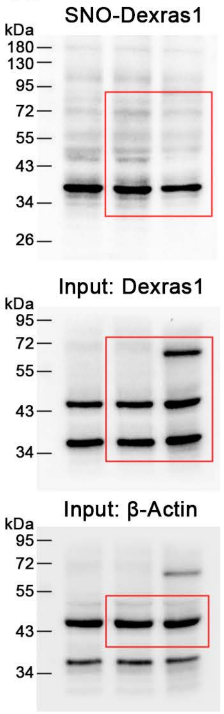

Figure 4B

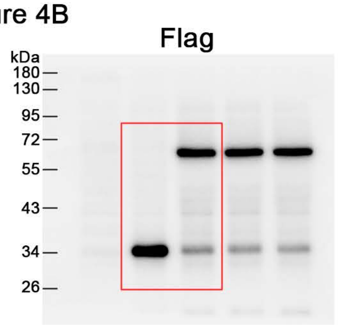

Figure 4C

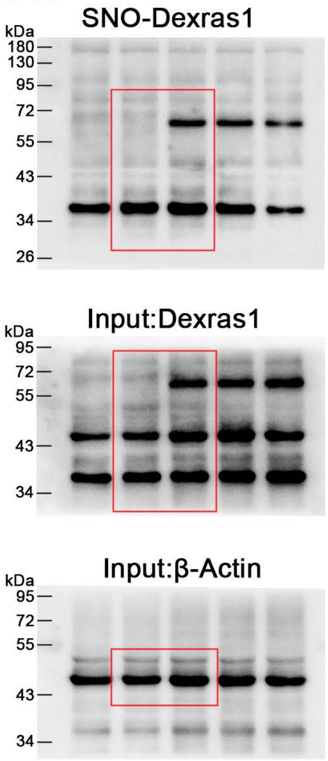

Figure S1

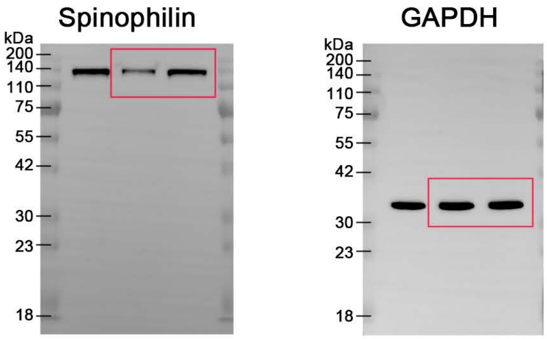

Supplement: Supplementary file 2 — Data S1. [file CNS-31-e70199-s001.pdf]
